# The Diagnostic Association of Radiological and Clinicopathological Parameters in Community-Acquired Pneumonia in Children: A Cross-Sectional Study

**DOI:** 10.7759/cureus.53626

**Published:** 2024-02-05

**Authors:** Yumna Asmaa, Spenta Kakalia, Muhammad Irtza, Rahat Malik

**Affiliations:** 1 Department of Paediatrics, Combined Military Hospital Lahore, Lahore, PAK; 2 Department of Paediatrics, Combined Military Hospital Lahore Medical College, Lahore, PAK; 3 Medical College, Services Institute of Medical Sciences Lahore, Lahore, PAK; 4 Department of Paediatrics, Combined Military Hospital Lahore Medical College and Institute of Dentistry, Lahore, PAK

**Keywords:** diagnosis, child, peadiatrics, pakistan, who- world health organization, hospital management, guidelines, c –reactive protein (crp), chest x-ray (cx-ray), pneumonia

## Abstract

Background

Globally, pneumonia is one of the significant causes of death in children under the age of five years. Assessment of disease severity is essential for clinical decision-making. Clinicians in resource-limited settings use the WHO Integrated Management of Childhood Illness (IMNCI) guidelines to diagnose and treat pneumonia. Chest X-rays and blood biomarkers are frequently performed in children presenting with pneumonia, but their role in clinical decision-making is limited.

Objective

To evaluate the association of chest X-ray results, clinical parameters, and blood inflammatory biomarkers with the severity of community-acquired pneumonia (CAP) in children to decide which tests are helpful in accurately classifying the severity of pneumonia.

Methods

This cross-sectional, analytical study was conducted at the Combined Military Hospital, Lahore, among 421 children aged two months to five years who were admitted with complaints of cough and difficulty breathing and were COVID-19 negative. Data was collected through a structured questionnaire, including demographic information and clinical categorization of pneumonia severity using WHO criteria, SpO_2_ levels, chest X-rays, complete blood count (CBC), and C-reactive protein (CRP) levels obtained within 24 hours of admission. Statistical evaluation of 323 children was done using SPSS version 26, and analysis of variance (ANOVA), chi-square test, and Fisher's exact test were applied to determine statistical significance. p-Value <0.05 was considered significant.

Results

The median age of the study population was eight months (IQR: 3-20 months); 113 (33.1%) were girls and 127 (37.2%) were underweight children. Eighteen (5.3%) patients had no pneumonia, 245 (71.8%) patients had non-severe pneumonia, and 78 (22.9%) patients had severe pneumonia. The clinical features of severe pneumonia were more common in children with radiologic findings of alveolar CAP than non-alveolar CAP (36.2% and 20%, respectively, p: 0.05). A higher percentage of patients with alveolar CAP had CRP >6 mg/dL than non-alveolar CAP (69.9% and 35%, respectively, p < 0.001). Patients with undernutrition (WAZ <-2 SD), hypoxemia (SpO_2 _<95%), and having CRP >6 mg/dL were associated with clinical features of severe pneumonia (46.1% vs. 33.8%, 100% vs 47.3%, and 67.9% vs 48.5%, respectively, p < 0.05). A significantly greater frequency of a bilateral multifocal distribution (p = 0.020), and the involvement of the right paracardiac region (p = 0.043) and the left lower lobe (p = 0.007) in those with severe pneumonia was observed.

Conclusion

Clinical diagnosis of pneumonia, along with the assessment of risk factors, including undernutrition and hypoxemia, should be adequate to diagnose pneumonia in children. Chest X-rays and CRP levels can be helpful in hospitalized children for whom physicians have difficulty deciding about antibiotic prescriptions, but their role in routinely classifying the severity of pneumonia in children is limited.

## Introduction

Globally, pneumonia is one of the significant causes of death in children under the age of five years. In 2015, approximately 700,000 children younger than five years died from pneumonia worldwide, despite general improvements in living conditions, improved nutrition, and better vaccines [1]. An estimated 14% of deaths in children under the age of five years are attributable to pneumonia [2]. According to the United Nations International Children's Emergency Fund (UNICEF) [MOU1], a child dies from pneumonia every 39 seconds [3]. In Pakistan, according to the 2017 Demographic and Health Survey, in the two weeks before conducting the survey, 14% of children under the age of five years had an acute respiratory infection [4]. Community-acquired pneumonia (CAP) is a common severe infection in children presenting in emergency department (ED) visits and hospitalizations. Assessment of disease severity is essential for clinical decision-making. Although the site-of-care decision is one of the most important in managing CAP, no validated prognostic tools can assist with this determination [5].

Clinicians in resource-limited settings use the World Health Organization (WHO) Integrated Management of Childhood Illness (IMNCI) guidelines to diagnose and treat pneumonia. The guidelines use fast breathing, or tachypnea, to diagnose mild-moderate pneumonia and chest indrawing to diagnose severe pneumonia [6]. Although, recent analyses suggest that the sensitivity (54%-62%) and specificity (59%-64%) of tachypnoea and sensitivity (38%-48%) and specificity (72%-80%) of lower chest indrawing are lower than initially estimated [7], indicating the requirement of laboratory parameters to aid clinical decision making. Several studies have assessed the utility of non-specific inflammatory biomarkers such as C-reactive protein (CRP), an acute-phase reactant released in response to cytokine interleukin-6, white cell count, and absolute neutrophil count (ANC) to discriminate probable bacterial infections from non-bacterial infections and to assess the severity of illness [8].

Chest radiograph is frequently performed in children presenting with pneumonia but usually does not affect the clinical outcome. In epidemiological studies, we observe the routine use of chest X-rays (CXRs) for classifying pneumonia. However, variability in its interpretation of the diagnosis of pneumonia in children is a recognized problem. Therefore, the WHO suggested standardized radiological definitions of pneumonia to allow more accurate comparative data in epidemiological studies to assess the impact of pneumococcal vaccination [9].

The purpose of this study is to evaluate the association of CXR findings, clinical parameters, and blood inflammatory biomarkers with the severity of CAP in children to decide whether CXRs and inflammatory biomarkers are helpful in accurately diagnosing the severity of pneumonia.

## Materials and methods

Study design and participants

The study was conducted at the Combined Military Hospital (CMH) Lahore's Pediatrics Inpatient Department for two years (from November 1, 2021, to December 31, 2023), using a descriptive, cross-sectional study design and non-probability sampling technique. All children aged two months to five years admitted to the Pediatric ward or ICU at the CMH, Lahore, with complaints of cough and difficulty breathing, who were COVID-19 negative (with no history of contact with patient having COVID-19, and confirmed by SARS-COV-2 PCR analysis) were included. Patients with recurrent pneumonia or severe chronic underlying comorbidities, including cystic fibrosis, swallowing dysfunction, immunodeficiency, congenital heart diseases, neurological diseases, or malformations, are excluded. The data was collected by researchers and appointed data collectors who were not involved in direct patient care; thus, the exercise did not interfere with routine patient care or pose an apparent additional risk to patients. Approval from the ethical committee has been obtained from CMH Lahore Medical College.

Procedure

We calculated a sample size of 385 patients using Raosoft® software, keeping the confidence interval at 95% with the margin of error at 5%. Sampling days were scheduled randomly once per week. Data was collected from all children meeting the inclusion criteria on the scheduled sampling day to avoid sampling bias. We collected data through a structured printed questionnaire after getting verbal informed consent from the parents/caregivers. The questionnaire included demographic information and clinical categorization of pneumonia severity using WHO criteria (Table 1) [10].

**Table 1:** WHO 2013 guidelines for managing children aged 2-59 months with cough, difficulty breathing, or both. Source: Ref. [10].

Classification	Clinical signs
Severe pneumonia	Any one of the following: SpO_2_ <90%, central cyanosis, severe respiratory distress, inability to breastfeed or drink or vomiting after every feed, altered consciousness, and convulsions
Pneumonia	Any one of the following: lower chest wall indrawings, fast breathing (respiratory rate >50 breaths per minute if aged 2-11 months, >40 breaths per minute if aged 12-60 months)

We used a modified Likert scale to quantify the severity of the symptoms and radiological and pathological findings. Detailed examination findings, including vitals and SpO_2_, were recorded, and we collected CXRs, complete blood count (CBC), and CRP levels within 24 hours of admission. Digital X-ray imaging equipment was used to obtain CXRs, and a single radiologist interpreted them according to WHO-SICR criteria (Table 2) [11].

**Table 2:** Definitions from the World Health Organization-defined standardized interpretation of pediatric frontal chest radiographs in pneumonia epidemiological studies ^a^The choice of the term "endpoint" refers to this being the endpoint of interest for trials of bacterial vaccines against pneumonia. ^b^"Portion of a lobe" means an opacity with a minor diameter greater than or equal to the size of a posterior rib and one adjacent rib space at the same level as the opacity. Where the opacity is irregular in shape (e.g., wedge-shaped), use the largest short-axis diameter (the largest diameter perpendicular to the line of the largest diameter of the opacity). ^c^In the presence of any visible adjacent opacity, a silhouette sign, where the length of loss of an anatomical border is greater than or equal to the size of a posterior rib and one adjacent rib space at the same level, is considered to show consolidation. A silhouette sign of this size without a visible adjacent opacity is considered the other infiltrate. ^d^Refers to the presence of these conclusions in the opinion of a panel of trained readers using the available World Health Organization-defined reference materials and methods. Source: Ref. [11].

Classification of findings	Endpoint consolidation^a^	A dense or confluent opacity that occupies a portion^b^ or whole of a lobe or entire lung may or may not have air bronchograms^c^
	Other infiltrates	It includes the linear and patchy opacities (interstitial infiltrate) in a lacy pattern featuring peribronchial thickening and multiple areas of atelectasis. It also includes minor patchy infiltrates that are insufficient to constitute endpoint consolidation and small areas of atelectasis that may be difficult to distinguish from consolidation in children.
	Pleural effusion	The presence of fluid in the lateral pleural space between the lung and chest wall that is spatially associated with a pulmonary parenchymal infiltrate (including other infiltrates) or has obliterated enough of the hemithorax to obscure any infiltrate; in most cases, this will be seen at the costo-phrenic angle or as a layer of fluid adjacent to the lateral chest wall; this does not include fluid seen in the horizontal or oblique fissures.
Conclusion^d^	Alveolar CAP	The presence of consolidation or pleural effusion, as defined above.
	Non-alveolar CAP	The presence of other (non-consolidation) infiltrates, as defined above, in the absence of a pleural effusion.
	Clinical CAP	Absence of consolidation, other infiltrates, or pleural effusion.

IBM SPSS Statistics for Windows, Version 26.0 (released 2019. IBM Corp., Armonk, NY) was used for data analysis. In reporting this study, we followed the "Strengthening the Reporting of Observational Studies in Epidemiology" (STROBE) guidelines.

Outcome measures

Our study's primary outcome was the proportion of patients with clinically severe pneumonia, with positive laboratory findings or CXRs indicative of alveolar pneumonia.

Statistical analysis

**Figure 1:**
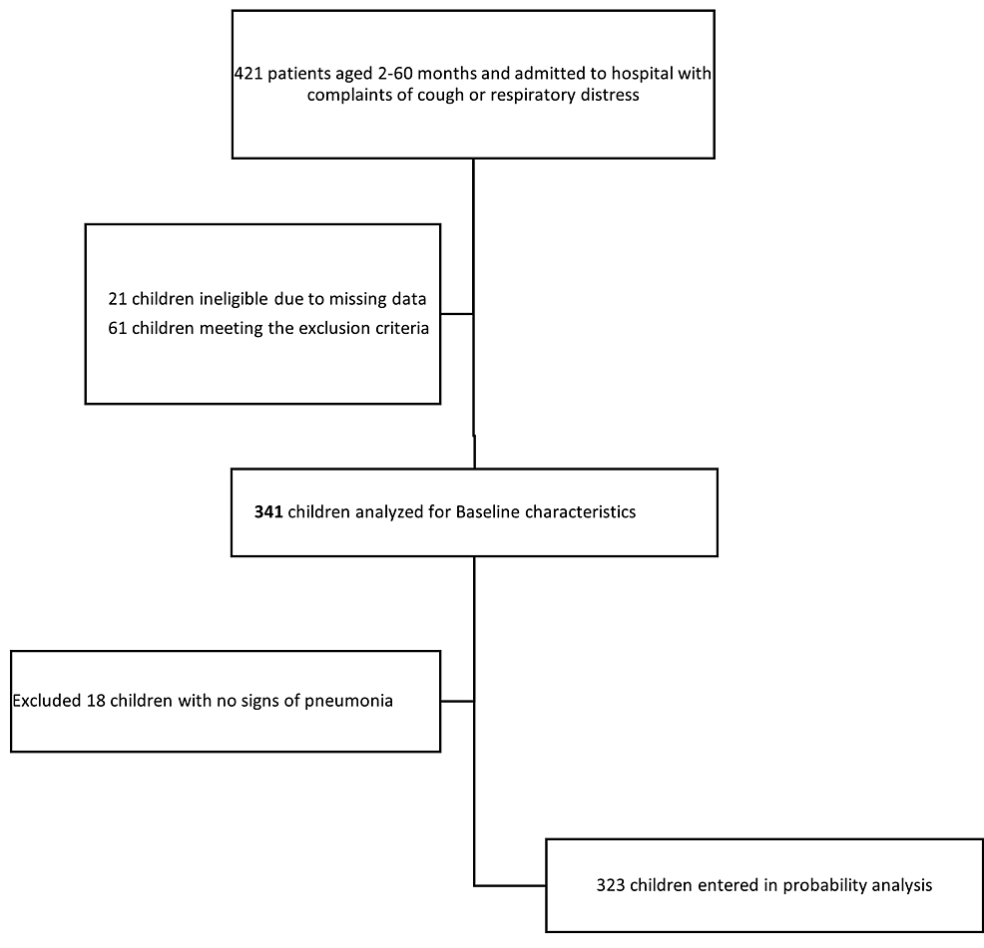
Study profile of children included in statistical analysis

Of the 421 patients analyzed, 61 children with exclusion criteria and 21 with missing data were excluded (Figure 1). The descriptive analysis of 341 children included mean age with SD, frequencies of nutritional status, clinical severity, SpO_2_, WBC count, ANC, CXR findings, and mean CRP value.

For further analyses, we excluded 18 children with no signs of pneumonia (Figure 1). In 323 patients with pneumonia and severe pneumonia, we calculated the association between clinical, pathological, and radiological parameters using ANOVA, chi-square, and Fisher's test. All the factors mentioned above were analyzed, and the results were combined.

## Results

Three hundred forty-one children from pediatric OPD were included in the analysis of baseline characteristics that are summarized in Table 3.

**Table 3:** Baseline characteristics of patients ^a^Data are median (IQR), mean (SD), or n (%). ^b^Defined as per WHO guidelines (weight for age <-3SD) (calculated WAZ using WHO child growth standards for children). IQR: interquartile range; SD: standard deviation, WAZ: weight-for-age Z score; CAP: community-acquired pneumonia; CRP: C-reactive protein; WBC: white blood cell; SpO_2_: saturation of peripheral oxygen.

Parameters	Proportion of patients n (%)^a^
General characteristics	
Age (months) [Median (IQR)]	8 (3-20)
Underweight^b^	127 (37.2%)
Gender	
Male	228 (66.9%)
Female	113 (33.1%)
Clinical condition	
No pneumonia	18 (5.3%)
Pneumonia	245 (71.8%)
Severe pneumonia	78 (22.9%)
SpO_2_	
<95%	176 (51.6%)
95% and above	165 (48.4%)
WBC count	
<4x10^9^	5 (1.5%)
4-10x10^9^	129 (37.8%)
>10x10^9^	207 (60.7%)
Mean CRP (mg/L) (SD)	20.3 (32.3)
Absolute neutrophil count	
<1500	10 (2.9%)
1500 and above	331 (97.1%)
Chest X-ray findings	
Clinical CAP	190 (55.7%)
Other infiltrates	27 (7.9%)
Primary endpoint pneumonia	113 (33.1%)
Atelectasis	31 (9%)
Interstitial changes	6 (1.7%)
Pleural effusion	2 (0.5%)

The median age of the study population was eight months (IQR: 3-20 months) (Table 3); 113 (33.1%) were girls and 127 (37.2%) were underweight children. We assigned pneumonia severity categories to patients based on the WHO 2013 classification (Table 1). Eighteen (5.3%) patients had no pneumonia, 245 (71.8%) patients had non-severe pneumonia, and 78 (22.9%) patients had severe pneumonia. SpO_2_ of <95% was seen in 176 (51.6%) patients. Leukocytosis (defined as a white blood cell count of more than 10 x 10^9^) was seen in 207 (60.7%) patients. The mean CRP value was 20.3. Normal ANC was found in 331 (97.1%) patients.

The clinical features of severe pneumonia were more common in children with alveolar CAP than non-alveolar CAP (36.2% and 20%, respectively, p: 0.001). Additionally, a higher percentage of patients with alveolar CAP had CRP >6 mg/dL than non-alveolar CAP (69.9% and 35%, respectively, p < 0.001). However, more patients with non-alveolar CAP had SpO_2_ <95% than patients with alveolar CAP (80% and 68.1%, respectively, p < 0.05%) (Table 4). Patients with undernutrition (WAZ < -2 SD), hypoxemia (SpO_2 _< 95%), and having CRP >6 mg/dL were associated with clinical features of severe pneumonia (46.1% vs. 33.8%, 100% vs 47.3%, and 67.9% vs 48.5%, respectively, p < 0.05) (Table 5).

**Table 4:** Demographic, clinical, and laboratory parameters of patients <5 years with radiographic presentations of CAP ^a^p-Values were calculated using a two-sided chi-square or Fisher's test, as appropriate. ^b^Frequency of patients in each radiological category. ^c^Defined as per WHO guidelines (weight for age <-3SD) (calculated WAZ using WHO child growth standards for children). CAP: community-acquired pneumonia; CRP: C-reactive protein; SpO_2_: saturation of peripheral oxygen; WAZ: weight-for-age Z score.

	Alveolar CAP (%)	Non-alveolar CAP (%)	Clinical CAP (%)	p-Value^a^
N (%)^b^	113 (34.9%)	20 (6.1%)	190 (58.8%)	
Underweight (n = 119)^c^	42 (37%)	7 (35%)	70 (36.8%)	0.983
Gender				
Male (n = 216)	79 (69.9%)	11 (55%)	126 (66.3%)	0.415
Female (n = 107)	34 (30%)	9 (45%)	64 (33.6%)	
Clinical severity				
Pneumonia (n = 245)	72 (63.7%)	16 (80%)	157 (82.6%)	0.001
Severe pneumonia (n = 78)	41 (36.2%)	4 (20%)	33 (17.3%)	
SpO_2_ <95% (n = 194)	77 (68.1%)	16 (80%)	101 (53.1%)	0.006
Leucocytosis (n = 194)	70 (61.9%)	15 (75%)	109 (57.3%)	0.272
CRP >6 mg/L (n = 172)	79 (69.9%)	7 (35%)	86 (45.2%)	0.000
Absolute neutrophil count <1500 (n = 10)	3 (2%)	0	7 (3.6%)	0.630

**Table 5:** Association between CAP severity and clinicopathological parameters ^a^p-Values were calculated using a two-sided chi-square or Fisher's test, as appropriate. ^b^Frequency of patients in each clinical category. ^c^Defined as per WHO guidelines (weight for age <-3SD) (calculated WAZ using WHO child growth standards for children). SpO_2_: saturation of peripheral oxygen; WBC: white blood cell count; CRP: C-reactive protein; WAZ: weight-for-age Z score.

Clinicopathological parameter	Pneumonia (%)	Severe pneumonia (%)	p-Value^a^
N (%)^b^	245 (75.8%)	78 (24.2%)	
Underweight (n = 119)^c^	83 (33.8%)	36 (46.1%)	0.050
Gender			
Male (n = 216)	162 (66.1%)	54 (69.2%)	0.611
Female (n = 107)	83 ((33.9%)	24 (30.8%)	
SpO_2_ <95% (n = 194)	116 (47.3%)	78 (100%)	0.000
WBC >10x10^9^ (n = 194)	142 (57.9%)	52 (66.6%)	0.221
CRP >6 mg/L (n = 172)	119 (48.5%)	53 (67.9%)	0.003
Absolute neutrophil count <1500 (n = 10)	9 (3.6%)	1 (1.2%)	0.288

The most frequently observed radiological presentation was focally distributed parenchymal densities (61, 54%), whereas 52 patients (46%) showed multifocal consolidations, predominantly unilaterally (32, 28.3%). Atelectasis and interstitial changes were detected in 31 (23.3%) and six patients (4.5%), respectively, and only two radiographs (1.5%) showed pleural effusions. Parenchymal densities were more observed in the right than the left lung (88 vs. 44), and consolidations were more frequent in the upper lung than in the middle and lower areas (65 vs 47 and 57). The most frequently affected locations were the right upper lobe (55, 48.7%), the right paracardiac field (44, 38.9%), the right lower lobe (38, 33.6%), and the left lower lobe (30, 26.5%) (Table 6).

**Table 6:** Association between CAP severity and radiological findings ^a^p-Values were calculated using a two-sided chi-square or Fisher's test, as appropriate. ^b^Location of consolidation on chest X-ray. ^c^Radiological findings other than consolidation. CAP: community-acquired pneumonia.

Distribution (n)	Pneumonia (%)	Severe pneumonia (%)	p-Value^a^
All locations (n = 113)^b^	72 (63.7%)	41 (36.2%)	
Focal (n = 61)	45 (62.5%)	16 (39%)	0.020
Unilateral multifocal (n = 32)	19 (26.3%)	13 (31.7%)	
Bilateral multifocal (n = 20)	8 (11.1%)	12 (29.2%)	
2 locations (n = 31)	18 (25%)	13 (31.7%)	0.282
3 locations or more (n = 21)	9 (12.5%)	12 (29.2%)	
Pattern^c^			
Atelectasis (n = 31)	21 (67.7%)	10 (32.2%)	0.832
Interstitial changes (n = 6)	2 (33.3%)	4 (66.7%)	0.082
Pleural effusion (n = 2)	2 (100%)	0	0.308
Distribution of consolidations/parenchymal densities			
Right upper lobe (n = 55)	32 (44.4%)	23 (56%)	0.233
Right hilum (n = 12)	9 (12.5%)	3 (7.3%)	0.390
Right paracardiac (n = 44)	23 (31.9%)	21 (51.2%)	0.043
Right lower lobe (n = 38)	23 (31.9%)	15 (36.5%)	0.616
Left upper lobe (n = 20)	11 (15.2%)	9 (21.9%)	0.371
Left hilum (n = 2)	1 (1.3%)	1 (2.4%)	0.684
Left paracardiac (n = 6)	2 (2.7%)	4 (9.7%)	0.112
Let lower lobe (n = 30)	13 (18%)	17 (41.4%)	0.007
Right lung (n = 88)	58 (80.5%)	30 (73.1%)	0.363
Left lung (n = 44)	22 (30.5%)	22 (53.6%)	0.015
Upper lung (n = 65)	40 (55.5%)	25 (60.9%)	0.575
Middle lung (n = 47)	24 (33.3%)	23 (56%)	0.018
Lower lung (n = 60)	33 (45.8%)	27 (65.8%)	0.040

Table 6 summarizes the associations between pneumonia severity and the radiological findings. In comparison with the children with pneumonia, we observed a significantly greater frequency of a bilateral multifocal distribution (p = 0.020), and the involvement of the right paracardiac region (p = 0.043) and the left lower lobe (p = 0.007) in those with severe pneumonia. There is also an association between the involvement of the left lung (p = 0.015) and the middle and lower lung fields (p = 0.018 and 0.040, respectively) and severe pneumonia.

## Discussion

In this study, we tried to determine the association of clinical diagnostic criteria set out by WHO 2013 guidelines for managing pneumonia in children under five years [12]. Our study included 245 children with pneumonia and 78 children with severe pneumonia as defined by WHO classification (Table 1). The most significant finding of our study was that following the clinical diagnostic criteria set out by WHO to diagnose pneumonia in children, only 18 of the 341 children admitted with cough as a primary complaint met the criteria for no pneumonia. WHO has defined clinical parameters for low-income countries (LICs) and middle-income countries (MICs) to diagnose pneumonia. However, we may need to consider other risk factors like hypoxemia, young age, and undernutrition, in addition to the WHO criteria to consider hospitalization in children with non-severe pneumonia [10].

Severe pneumonia was significantly associated with underweight children. In Pakistan, 23% of children under the age of five years are underweight, and 8% are severely underweight [4]. Many studies have demonstrated the association of low weight and malnutrition with pneumonia in children [10,13]. Malnutrition can be a factor for increased hospitalizations in children with pneumonia, who, according to WHO 2013 guidelines, can be managed with oral antibiotics at home. Given the high proportion of malnourished children in Pakistan and the pneumonia, the government and other stakeholders should invest more healthcare funds to work on both these issues together.

Our study found that hypoxemia (SpO_2 _< 95%) is significantly associated with severe pneumonia (100%, p < 0.001). Rees et al. and Karim et al. also discovered a significant association of hypoxemia with radiographic pneumonia [7] and radiological pneumonia [14]. SpO_2_ is a non-invasive and cheap parameter that can help identify high-risk patients presenting with pneumonia.

In a child admitted with complaints of cough and respiratory distress, chest radiographs are routinely done along with blood tests to confirm a clinical diagnosis of pneumonia. Chest radiographs pose a few issues. First, in a resource-poor country like Pakistan, chest radiographs are not available to all children. Secondly, radiographs expose patients to radiation, which can have long-lasting effects. Finally, in a country with limited healthcare funds, CXRs and other hematological investigations add expense to an already stretched system, especially if their role in altering management is limited. This study explores the relevance of commonly utilized diagnostic modalities among children with WHO-defined pneumonia at a tertiary care hospital in Pakistan with clinical severity of pneumonia. Patients with severe pneumonia and having radiological findings of alveolar CAP had a significantly higher CRP (53 of 78 [67.9%] and 79 of 113 [69.9%], respectively), and this has also been borne out in other studies [15]. The level of CRP is directly correlated with bacterial pneumonia, helping determine antibiotic use [16-18]. This could signify that CRP and clinical features might be beneficial in determining prognosis in hospitalized patients with severe pneumonia [5].

Traditionally, physicians use CXRs to make a final diagnosis of pneumonia. In a study conducted in Peshawar, Pakistan, only 64.4% of children meeting the clinical diagnosis of pneumonia had CXR findings, and tachypnea was the most sensitive indicator of pneumonia [14]. A similar study demonstrated 82% of normal CXRs in children with non-severe pneumonia, using WHO definitions [19]. Va de Maat et al. studied patients one to five years old in the emergency room with fever and cough, followed by a one-week follow-up. Although patients with CXRs were more likely to initially receive an antibiotic, abnormal findings on CXRs were not associated significantly with antibiotic use [20]. This supports our findings that CXRs may not be necessary for the diagnosis of pneumonia, and the clinical criteria alone are robust enough to make the diagnosis.

CXRs of children with pneumonia have a myriad of findings. The WHO has categorized pneumonia based on the presentation of CXRs [11]. A few studies have explored the clinical significance of different patterns of pneumonia and found that alveolar CAP suggests bacterial etiology [21]. In our study, 72 patients (63.7%) with pneumonia had a statistically significant finding of alveolar CAP on CXR, while only 41 (36.2%) with a clinical diagnosis of severe pneumonia had alveolar CAP. In this study, the patients with severe pneumonia, compared with those with pneumonia, were more likely to have bilateral multifocal distribution, right paracardiac and left lower lobe distribution, and left and middle lung distribution. In a study of children, Semernik et al. found that the most common findings were right-sided and focal or segmental [22]. One hundred and fifty-seven of 245 (82.6%) children admitted with pneumonia and 33 of 78 (17.3%) children with severe pneumonia had the normal appearance of CXRs, which can be associated with early presentation of disease, increased incidence of viral etiology [23,24], or inadequacy of WHO 2013 guidelines in differentiating patients with pneumonia from other etiologies (like bronchitis or bronchiolitis) which present with wheeze that does not improve with two doses of short-acting bronchodilators, as suggested [19].

To the best of our knowledge, this is the first study done in an LIC that evaluated the use of CXRs and CRP in children admitted with pneumonia and compared its usefulness with WHO diagnostic guidelines. We included all children admitted with primary cough or respiratory distress complaints, accurately reflecting the population of children presenting in the hospital. We included children who tested COVID-19 negative, and had no history of contact with cases of COVID-19 pneumonia, to ascertain the burden of non-COVID pneumonia as we are uncertain about applying WHO 2013 guidelines on children presenting with COVID-19 pneumonia.

Our study has several limitations. First, it is a single-center study that includes only children without comorbidities, which may affect the generalizability of our results to hospitals in other LICs. As this is a cross-sectional study, we were unable to evaluate the association of the severity of pneumonia and diagnostic evaluation with the disease outcome, and we were unable to assess the sensitivity and specificity of the investigations or their prognostic value. We analyzed the radiologist's interpretation of CXR only and did not consider the inter-observer variability, a well-recognized limitation of CXR. We did not consider the factors that influence the biomarker concentrations, including duration of illness, prior use of antibiotics, and malnutrition. In our study, only two children had pleural effusion at presentation, so we could not assess the association of biomarkers with complications of pneumonia. We did not consider the vaccination status of children in our study.

## Conclusions

Clinical guidelines for diagnosing pneumonia and associated risk factors (like undernutrition and hypoxemia) should be adequate to diagnose pneumonia in children. In Pakistan, all primary healthcare workers should be well-versed with the clinical diagnostic criteria for pneumonia, which will help decrease the disease burden. CXRs and CRP levels can be helpful in hospitalized children for whom physicians have difficulty deciding about antibiotic prescriptions.
